# Protection of bovine mammary epithelial cells against lipopolysaccharide-induced inflammatory responses using *Centella asiatica* through its antioxidant and anti-inflammatory activities

**DOI:** 10.5713/ab.25.0089

**Published:** 2025-08-12

**Authors:** Do Hyun Kim, Hyuk Cheol Kwon, Jong Hyeon Han, Hyun Su Jung, Dong-Min Shin, Sung Gu Han

**Affiliations:** 1 Department of Food Science and Biotechnology of Animal Resources, Konkuk University, Seoul, Korea; 2 Department of Food Science and Technology, Keimyung University, Daegu, Korea

**Keywords:** Bovine Mastitis, Centella Asiatica, Ethanol Extract, MAC-T Cell, Oxidative Stress, Phytogenic Feed Additive

## Abstract

**Objective:**

Bovine mastitis, an inflammatory condition affecting dairy cow udders, results in decreased milk quantity and quality, posing significant economic losses in the dairy industry. With increasing interest in natural products, *Centella asiatica* has garnered attention for its potent anti-inflammatory and antioxidant properties. This study aimed to investigate the potential protective effects of *C. asiatica* extracts (CE) against lipopolysaccharide (LPS)-induced inflammation in bovine mammary epithelial cells (MAC-T).

**Methods:**

CEs were prepared by extracting *C. asiatica* leaf powder using ethanol at concentrations of 60%, 70%, 80%, 90%, and 100%. In LPS-stimulated MAC-T cells, the study investigated the ability of CE to reduce oxidative stress and inflammation and its impact on casein protein synthesis.

**Results:**

CE with 60% ethanol (CE60) exhibited the highest radical scavenging activity and total phenolic content among the tested extracts. In MAC-T cells, CE60 significantly attenuated LPS-induced inflammation by down-regulating pro-inflammatory cytokines such as cyclooxygenase-2, tumor necrosis factor-α, interleukin (IL)-1β, and IL-6, as well as inhibiting nuclear factor kappa B activation. CE60 also reduced intracellular reactive oxygen species through upregulating nuclear factor erythroid-2-related factor 2 and associated antioxidant enzymes, including glutathione peroxidase (GPx) 1, GPx4, superoxide dismutase (SOD) 1, SOD2, and catalase. Moreover, CE60 restored the synthesis of casein proteins (CSN1S1, CSN1S2, and CSN2) in LPS-treated MAC-T cells, indicating a protective effect on lactation function under inflammatory conditions.

**Conclusion:**

Taken together, CE60 has potential as a natural substance for the prevention of bovine mastitis by reducing oxidative stress and inflammation in mammary epithelial cells.

## INTRODUCTION

Bovine mastitis, an inflammatory response resulting from physical trauma or microbial infections in the mammary gland, can lead to significant economic loss in the dairy industry due to reduced milk yield and poor milk quality [1]. Especially, dairy cows in the periparturient period become vulnerable to mastitis as a result of reactive oxygen species (ROS) overproduction and immune dysfunction resulting from hormonal changes associated with milk production [2–4]. ROS are generated during physiological metabolism in mitochondrial respiratory chain of aerobic organisms and are neutralized through enzymatic cellular defense mechanisms and non-enzymatic antioxidants under homeostatic conditions [5]. However, excessive ROS-induced redox imbalance causes oxidative stress (OS) and damage of cellular constituent, including DNA, proteins, and lipids, ultimately leading to the impairment of mammary tissue [6]. Therefore, dietary supplementation with antioxidants for dairy cows can enhance milk productivity and quality by regulating ROS accumulation, supporting immune function, and preventing bovine mastitis [7].

*Centella asiatica*, a perennial herbaceous plant, holds significant importance in traditional Asian medicine for its various applications and disease treatments [8]. It is widely known for its antioxidant, anti-inflammatory, neuroprotective, and wound-healing properties, with the primary pharmacologically active constituents being triterpenes, such as asiaticoside, asiatic acid, madecassoside, and madecassic acid [9–11]. In a previous study, administering a standardized extract of *C. asiatica* (ECa233) to adult male Wistar rats resulted in the inhibition of lipid peroxidation in liver tissue by activating catalase (CAT) [12]. Also, oral treatment with a crude methanol extract of *C. asiatica* in lymphoma-bearing mice significantly increased the levels of antioxidant enzymes such as superoxide dismutase (SOD), CAT, and glutathione peroxidase (GPx) [13]. Furthermore, the 50% ethanol extract of *C. asiatica* exhibited antioxidant and anti-inflammatory activities through the inhibition of tumor necrosis factor-α (TNF-α), nitric oxide, and extracellular-signal-regulated kinase 1/2 (ERK1/2)-mediated nuclear factor kappa B (NF-κB) pathway in BV2 microglial cells treated with lipopolysaccharide (LPS) [14]. These previous studies suggest that *C. asiatica* may possess antioxidant and anti-inflammatory properties for the treatment and prevention of bovine mastitis in dairy cows.

Naturally occurring antioxidants, including plant-derived phytochemicals, have recently attracted considerable attention in animal nutrition [15]. Dietary phytochemicals are believed to directly affect specific molecular sites or indirectly influence molecular pathways, thereby improving animal health, performance, and product quality [15]. However, the protective effects of phytochemical-derived antioxidants against OS in the prevention of bovine mastitis have been rarely investigated. Indeed, previous studies have mainly investigated antimicrobial and anti-inflammatory activities of diverse phytochemicals against bovine mastitis [16,17]. In contrast, our current study investigated the antioxidant properties of *C. asiatica* for its potential to prevent bovine mastitis. Therefore, we aimed to optimize the extraction of *C. asiatica* leaves by identifying conditions that yield the highest total phenolic content (TPC) and radical scavenging activity. We then evaluated the protective effects of the resulting extract against OS, inflammatory responses, and impacts on milk productivity in MAC-T cells treated with LPS. To model bovine mastitis at the cellular level, we employed the MAC-T cell line, which had been immortalized through the introduction of the simian virus 40 large T-antigen [18].

## MATERIALS AND METHODS

### Materials

3-(4,5-Dimethylthiazol-2-yl)-2,5-diphenyltetrazolium bromide (MTT) was purchased from Amresco. 2′,7′-dichlorofluorescin diacetate (DCFH-DA), LPS from *Escherichia coli* O111:B4, progesterone, insulin, 2,2-diphenyl-1-picrylhydrazyl (DPPH), and 2,2′-azino-bis (3-ethylbenzothiazoline-6-sulfonic acid) (ABTS) were purchased from Sigma-Aldrich. Trypsin-etylenediaminetetraacetic acid (EDTA) solution, fetal bovine serum (FBS), penicillin/streptomycin, and Dulbecco’s modified Eagle medium (DMEM) were obtained from Welgene. TRIzol reagent was purchased from Life Technologies. Phosphate-buffered saline (PBS) was purchased from Lonza. Antibodies against cyclooxygenase-2 (COX-2) and nuclear factor erythroid-2-related factor 2 (Nrf2) were purchased from Abcam. Antibodies for goat anti-rabbit IgG-horseradish peroxidase (HRP), lamin B, NF-κB p65, and donkey anti-goat IgG-HRP were obtained from Santa Cruz Biotechnology. Antibodies against glyceraldehyde 3-phosphate dehydrogenase (GAPDH) were purchased from Sigma-Aldrich.

### Preparation of *Centella asiatica* extracts

*C. asiatica* leaf powder was obtained from a local farm. *C. asiatica* powder was mixed with various solvents, such as ethanol, at different concentrations (60%, 70%, 80%, 90%, and 100% ethanol, v/v). The solid (powder) to solvent ratio was 1:20, based on a previous study [19]. These mixtures were stirred for 16 h at room temperature (RT) and then filtered using Whatman No. 1 filter paper. The filtrate was evaporated using a rotary evaporator (Tokyo Rikakikai) under reduced pressure at 50°C. The water extract of *C. asiatica* was prepared as described in a previous study [20]. For the water extract, 25 g of the powder was blended with 500 mL of deionized water (DW). The mixture was boiled at 100°C with stirring for 30 min and then cooled to 60°C. Subsequently, the extracts were filtered through a Whatman No. 1 filter (GE Healthcare Life Science). All filtrates were freeze-dried and stored at −80°C until further use.

Prior to use in cell culture experiments, freeze-dried ethanol extracts of *C. asiatica* were dissolved in DW and filtered through a 0.45-μm filter (Millipore). The ethanol extracts of *C. asiatica* samples were named according to their solvent conditions: CE60 (extracted with 60% ethanol), CE70 (extracted with 70% ethanol), CE80 (extracted with 80% ethanol), CE90 (extracted with 90% ethanol), CE100 (extracted with 100% ethanol), and CW (extracted with DW).

### 2,2-Diphenyl-1-picrylhydrazyl, 2,2′-azino-bis (3-ethylbenzothiazoline-6-sulfonic acid)^+^ free radical scavenging activity

Radical scavenging assays were conducted following the methodology outlined in a previous study [20]. In the DPPH assay, an ethanolic DPPH solution (0.1 mM) was mixed with *Centella asiatica* extracts (CE) in a 96-well plate, and radical scavenging reactions were performed in the dark at RT for 30 min (n = 3 wells per group). DPPH solution in ethanol was used as a control. Optical density (OD) was measured at 515 nm using a spectrophotometer (BioTek Instruments). DPPH free radical scavenging activity (%) was calculated using the following formula:


(1) $$\%\;\text{Scavenging}=(1-[{\text{OD}}_{\text{sample}}/{\text{OD}}_{\text{control}}])\times 100\;(\%)$$

For the ABTS assay, the 14.8 mM of ABTS reagent was combined with potassium persulfate in a 1:1 ratio (v/v) and allowed to react in the dark for 16 h at RT. The resulting ABTS^+^ solution was then diluted with DW to achieve an OD_734 nm_ of 0.700±0.05. CE and ABTS^+^ solutions were mixed in a 96-well plate and radical scavenging reactions were carried out in the dark at RT for 15 min (n = 3 wells per group). DW containing ABTS^+^ solution was used as a control. The OD of the samples was measured at 734 nm using a spectrophotometer. ABTS^+^ free radical scavenging activity (%) was calculated using the following formula:


(2) $$\%\;\text{Scavenging}=(1-[{\text{OD}}_{\text{sample}}/{\text{OD}}_{\text{control}}])\times 100\;(\%)$$

### Determination of total phenolic contents in *Centella asiatica* extracts

The TPC in CE were determined using the Folin–Ciocalteu colorimetric method [21]. In brief, 30 μL of CE was diluted with 120 μL of DW in a 96-well plate (n = 3 wells per group). Subsequently, 20 μL of Folin–Ciocalteu phenol reagent and 30 μL of sodium carbonate (Na_2_CO_3_) were added and incubated for 30 min in the dark at RT. To prepare the standard curve, gallic acid at concentrations ranging from 10 to 60 μg/mL was used. OD was measured at 595 nm using a spectrophotometer. TPC of CE was expressed as gallic acid equivalents (GAE, μg GAE/mL).

### Cell culture and treatments

Immortalized bovine mammary epithelial cells, MAC-T were maintained in DMEM supplemented with 10% FBS, 1% penicillin/streptomycin, 1 μg/mL progesterone, and 5 μg/mL insulin and cultured in a CO_2_ incubator at 37°C. The cells were cultured until they reached 80%–90% confluence. Prior to LPS treatments, the cells were pre-treated with CE (6.25–400 μg/mL) for 12 h. Then, LPS (1 μg/mL) was added to the cells for 4–12 h. The selection of the LPS concentration and treatment duration was based on previous studies [22].

MAC-T cell differentiation was conducted following protocols from previous studies [23,24]. In summary, MAC-T cells were cultured to 90% confluence. Subsequently, the cells were serum-starved in serum-free DMEM for 16 h. Cells were then cultured in differentiation medium (low glucose DMEM containing 5% FBS, 1% penicillin/streptomycin, 5 μg/mL insulin, 5 μg/mL prolactin, 1 μM retinoic acid, and 1 μg/mL hydrocortisone) at 37°C. The medium was refreshed daily for 8 days. To assess casein mRNA expression, CE60 was added at concentrations of 25, 100, and 400 μg/mL during the final medium change. After 12 h, cells were treated with LPS for an additional 12 h before harvesting.

### 3-(4,5-Dimethylthiazol-2-yl)-2,5-diphenyltetrazolium bromide and lactate dehydrogenase release assay

Cell viability was assessed using the MTT assay, following the method described in a previous study [25]. Cells were seeded in a 96-well plate and treated with CE (6.25–400 μg/mL) for 24 h when the cells reached 80%–90% confluency (n = 3 wells per group). The OD of the samples was measured at 570 nm and 630 nm using a spectrophotometer.

The lactate dehydrogenase (LDH) release assay was performed using CytoTox 96 Non-Radioactive Cytotoxicity Assay kit (Promega) according to the manufacturer’s protocol. Briefly, cells were seeded in a 96-well plate and treated with CE (6.25–400 μg/mL) for 24 h when the cells reached 80%–90% confluency (n = 3 wells per group). Cell membrane lysis buffer was used to determine maximum LDH release. The OD of the samples was measured at 490 nm using a spectrophotometer.

### Western blot analysis

Western blot analysis was performed to evaluate the effects of CE on the expression of COX-2 and cell signaling proteins. MAC-T cells were seeded in 6-well plates and incubated until 80% confluence was reached. Subsequently, the cells were pre-treated with CE at concentrations of 25, 50, 100, and 400 μg/mL for 12 h, followed by exposure to LPS (1 μg/mL) for an additional 12 h (n = 3 wells per group). For cell lysate preparation, the cells were lysed in radioimmunoprecipitation (RIPA) buffer (ELPIS-BIOTECH), and 1 mM phenylmethanesulfonyl fluoride. Additionally, a 1% protease inhibitor cocktail (Abbkine) was added to the RIPA buffer. The lysates were centrifuged at 21,000×g at 4°C for 20 min. The supernatants were collected determined using the Pierce BCA Protein Assay Kit (Sigma-Aldrich). Protein samples for Western blot were separated using Sodium dodecyl sulfate (SDS)-polyacrylamide gel electrophoresis and transferred onto nitrocellulose membranes. Protein bands were visualized using an enhanced chemiluminescence detection reagent (Thermo Fisher Scientific) and quantified using ImageJ software. The protein levels were normalized to those of GAPDH or lamin B.

### Real-time polymerase chain reaction

To evaluate the gene expression of pro-inflammatory cytokines (COX-2 and antioxidant-related enzymes), total RNA was extracted from MAC-T cells (n = 3 wells per group) using a TRIzol reagent. Reverse transcription was conducted for cDNA synthesis using the TOPscript RT DryMIX kit (Enzynomics) according to the manufacturer’s instructions. To determine the mRNA expression levels, real-time polymerase chain reaction (RT-PCR) was conducted using the Roche LightCycler 96 system (Roche) and 2X Real-Time PCR mix (SolGent). The PCR cycles were executed under the following conditions: initial denaturation at 95°C for 15 min, followed by 40 cycles of denaturation at 95°C for 20 s, annealing at 58°C for 40 s, and extension at 60°C for 30 s. The mRNA expression levels were relatively quantified using the ΔΔCq method, and the GAPDH mRNA level was used as the internal control. Primers for the target genes were designed using AmplifX software and purchased from BIONICS. The specific primer sequences are listed in Supplement 1.

### Nuclear fractionation

The translocation of NF-κB p65 and Nrf2 into the cell nuclei was assessed by nuclear fractionation, as described previously [26]. Briefly, MAC-T cells were grown in 10-cm plates and then treated with CE60 of 25, 100, and 400 μg/mL. After 12 h, cells were exposed to 1 μg/mL LPS for 6 h (n = 3 wells per group). For nuclear fractionation, cells were harvested and lysed in a hypotonic buffer solution containing 20 mM Tris (pH 7.4), 10 mM sodium chloride (NaCl), 3 mM magnesium chloride, and protease inhibitor cocktails. Subsequently, 10% Triton X-100 was added to extract cytosolic proteins. The cell lysates were centrifuged at 650×g at 4°C for 10 min and the supernatants were collected as cytosolic fractions. The pellets obtained in the previous step were resuspended in nuclear extraction buffer containing 100 mM Tris (pH 7.4), 2 mM sodium orthovanadate, 100 mM NaCl, 1% Triton X-100, 1 mM EDTA, 10% glycerol, 0.1% SDS, 20 mM tetrasodium pyrophosphate, and protease inhibitor cocktails. The resuspended samples were centrifuged at 14,000×g at 4°C for 20 min. The supernatants were collected as the nuclear fraction and used for Western blot analysis.

### High-performance liquid chromatography analysis

An high-performance liquid chromatography (HPLC) system (Agilent 1200 series, Agilent) equipped with a Primesep 100 column (150 mm×4.6 mm i.d. and 5 μm particle size) was utilized. The flow rate of the mobile phase was maintained at 1 mL/min and controlled by quaternary gradient pumps at 40°C. The mobile phase comprised acetonitrile and DW. The injection volume for the sample was set at 10 μL, and the column temperature was maintained at 40°C. The samples were analyzed at a wavelength of 206 nm. The madecassoside, madecassic acid, asiaticoside, and asiatic acid contents of CE60 were determined by comparing the peak areas of the samples with those of the standards.

### Evaluation of reactive oxygen species level

To assess the ROS scavenging effect of CE60, intracellular ROS levels were measured using DCFH-DA reagent. Cells were seeded in 6-well plates and grown to 80% confluence. The cells were treated with CE60 at concentrations of 25, 100, and 400 μg/mL for 12 h, followed by an additional 4 h of LPS exposure (n = 3 wells per group). After treatment, the cell culture medium was replaced with fresh medium containing 1% FBS, 1% penicillin/streptomycin, 1 μg/mL progesterone, and 5 μg/mL insulin. Subsequently, the cells were incubated with 20 μM DCFH-DA at 37°C for 30 min in a 5% CO_2_ incubator. After rinsing with PBS, green fluorescence (DCFH-DA-positive) was visualized using an Eclipse Ti2-U fluorescence microscope (Nikon) and images were captured with a Nikon Eclipse Ts2R camera (Nikon). The intensity of green fluorescence was quantified using the ImageJ software. For additional analysis of ROS levels, cells incubated with 15 μM DCFH-DA were analyzed for ROS production using a CytoFLEX flow cytometer (Beckman Coulter). In each treatment group, 25,000 cells were counted, and DCFH-DA-positive cells were detected and analyzed using CytExpert software (Beckman Coulter).

### Evaluation of glutathione level

Glutathione (GSH) levels in MAC-T cells were determined using a Glutathione Fluorometric Assay Kit (Biovision) following the manufacturer’s protocol. MAC-T cells were seeded in 6-well plates and grown to 80% confluence. The cells were pre-treated with CE60 (25, 100, and 400 μg/mL) for 12 h, followed by LPS treatments (1 μg/mL) for 12 h (n = 3 wells per group). Cells were trypsinized and centrifuged at 700×g for 5 min. The cell pellets were incubated with lysis buffer at 4°C for 10 min. The cell lysates were centrifuged at 21,000×g at 4°C for 10 min and the supernatants were transferred to a 96-well plate. Monochlorobimane and glutathione S-transferase (GST) reagents were introduced to each well and incubated at 37°C for 1 h. The fluorescence intensity was then quantified using a SpectraMax Gemini EM Microplate Reader (Molecular Devices) with excitation and emission wavelengths set at 360 nm and 460 nm, respectively.

### Statistical analysis

Statistical analysis was performed using SPSS-PASW Statistics software version 22.0 for Windows (IBM). All experimental data are presented as mean±standard error of the mean from at least three independent biological replicates (n = 3). For the analysis of statistical significance, one-way analysis of variance with Tukey’s honestly significant difference post hoc test was used for multiple group comparisons, and Student’s t-test was applied for two-group comparisons. A p-value less than 0.05 was considered statistically significant.

## RESULTS

### Total phenolics and free radical scavenging properties of *Centella asiatica* extracts

**Figure 1:**
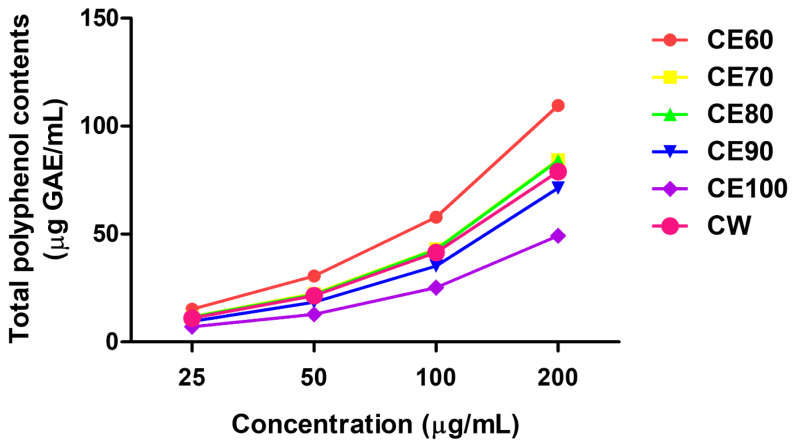
Total polyphenol contents (mg GAE/mL, TPC) of different solvent extracts of *Centella asiatica* (CE). The TPC of each extract was measured at various concentrations (25, 50, 100, and 200 μg/mL). All experiments were performed in triplicate (n = 3). The extracts obtained using different solvents were named as follows: CE60 (extracted with 60% ethanol), CE70 (extracted with 70% ethanol), CE80 (extracted with 80% ethanol), CE90 (extracted with 90% ethanol), CE100 (extracted with 100% ethanol), and CW (extracted with distilled water).

**Figure 2:**
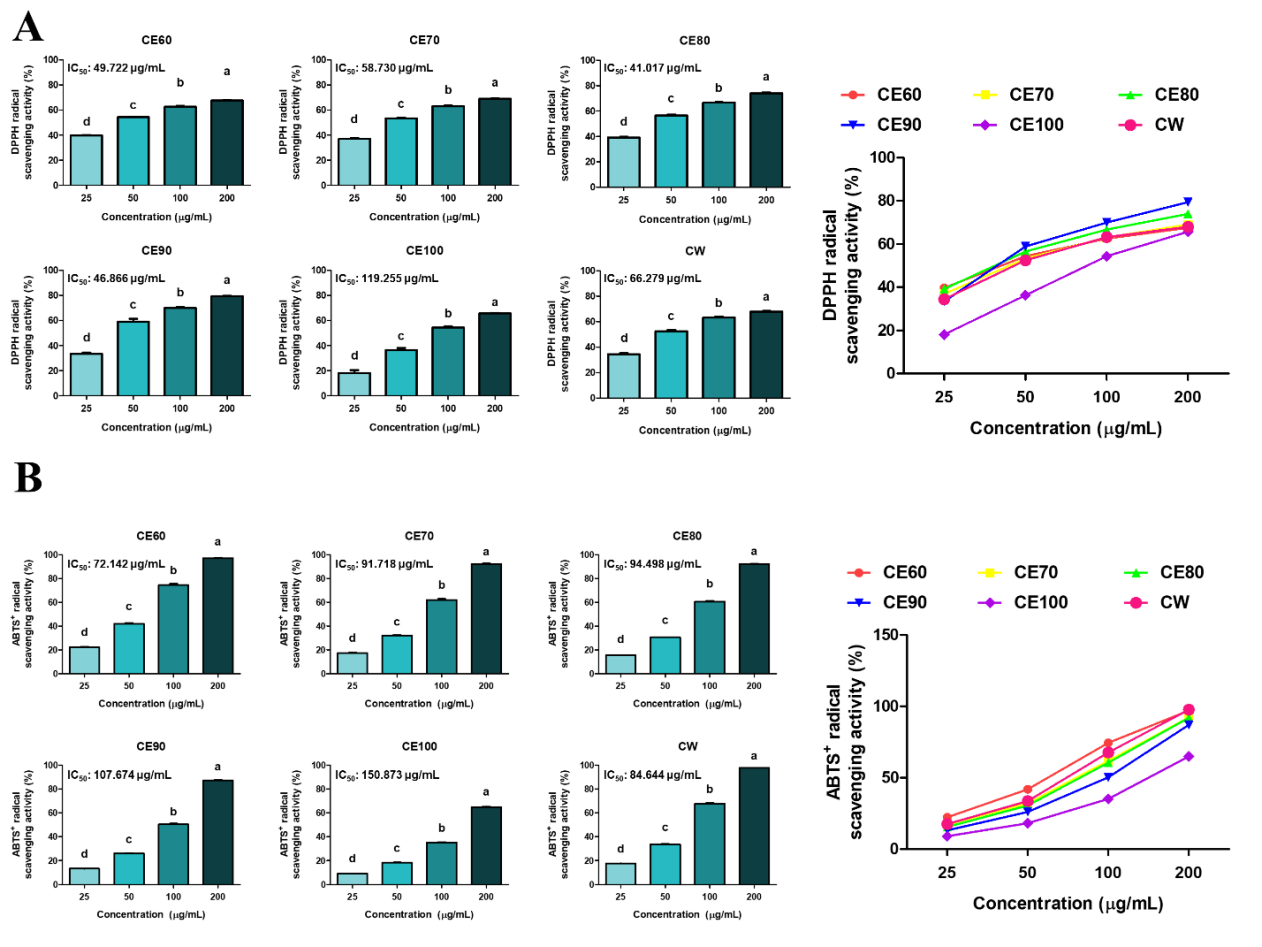
Free radical scavenging activities of different solvent extracts of *Centella asiatica* (CE). (A) DPPH and (B) ABTS^+^ free radical scavenging activities (%) of CE were measured at various concentrations (25, 50, 100, and 200 μg/mL). IC_50_ (μg/mL) values were calculated for each extract. All experiments were performed in triplicate (n = 3), and the data represent the mean±standard error. The extracts obtained using different solvents were named as follows: CE60 (extracted with 60% ethanol), CE70 (extracted with 70% ethanol), CE80 (extracted with 80% ethanol), CE90 (extracted with 90% ethanol), CE100 (extracted with 100% ethanol), and CW (extracted with distilled water). ^a–d^ Different letters represent statistical differences (p<0.05). DPPH, 2,2-diphenyl-1-picrylhydrazyl; ABTS, 2,2′-azino-bis (3-ethylbenzothiazoline-6-sulfonic acid).

TPC and free radical scavenging activity were evaluated to determine the optimal extraction condition for *C. asiatica* (Figures 1, 2). TPC increased proportionally with CE concentrations (25, 50, 100, and 200 μg/mL) (Figure 1). Of all the solvent conditions tested, CE60 at 200 μg/mL yielded the highest TPC, measuring 109.59 μg GAE/mL. However, when ethanol concentrations exceeded those used for CE60 at 200 μg/mL, the TPC began to decrease (84.46 μg GAE/mL for CE70, 84.03 μg GAE/mL for CE80, 78.99 μg GAE/mL for CW, 71.30 μg GAE/mL for CE90, and 49.25 μg GAE/mL for CE100).

DPPH and ABTS^+^ free radical scavenging activities were enhanced in a CE concentration-dependent manner (Figure 2). The IC_50_ value, defined as the concentration required to scavenge 50% of free radicals, was calculated to evaluate the antioxidant activity of CE. CE80 and CE60 showed the lowest IC_50_ values in DPPH and ABTS^+^ free radical scavenging activities, respectively. Based on these results, CE60, CE70, and CE80 were selected for subsequent experiments.

### Cytotoxicity of *Centella asiatica* extracts in MAC-T cells

**Figure 3:**
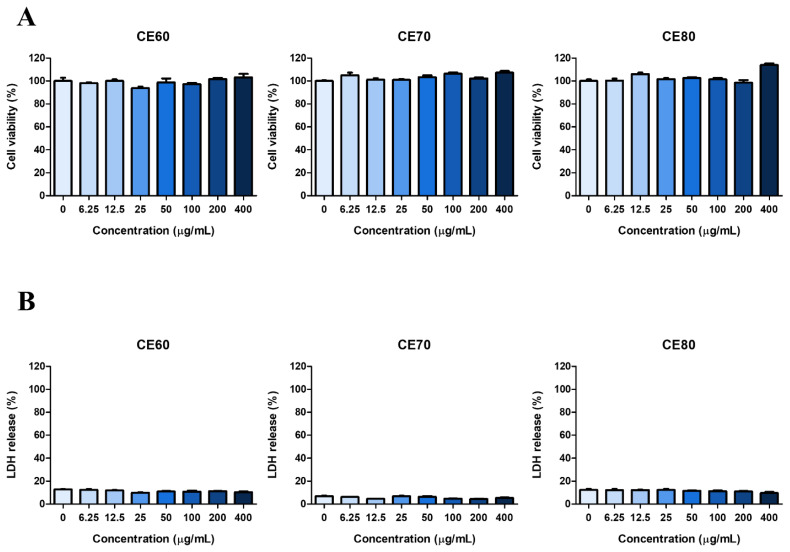
Cytotoxicity of different solvent extracts of *Centella asiatica* (CE) in MAC-T cells. (A) MTT assay and (B) LDH release assay were used to determine the cytotoxicity of CE. Cells were treated with various concentrations (0–400 μg/mL) of CE for 24 h. All experiments were performed in triplicate (n = 3), and the data represent the mean±standard error. The extracts obtained using different solvents were named as follows: CE60 (extracted with 60% ethanol), CE70 (extracted with 70% ethanol), and CE80 (extracted with 80% ethanol). MTT, 3-(4,5-Dimethylthiazol-2-yl)-2,5-diphenyltetrazolium bromide; LDH, lactate dehydrogenase.

The cytotoxicity of CE in MAC-T cells was assessed using MTT and LDH release assays (Figure 3). Treatment of cells with CE60, CE70, and CE80 at concentrations ranging from 6.25 to 400 μg/mL exhibited no cytotoxicity compared to the control. Therefore, CE concentrations up to 400 μg/mL were used in subsequent experiments.

### Anti-inflammatory effects of *Centella asiatica* extracts in MAC-T cells

**Figure 4:**
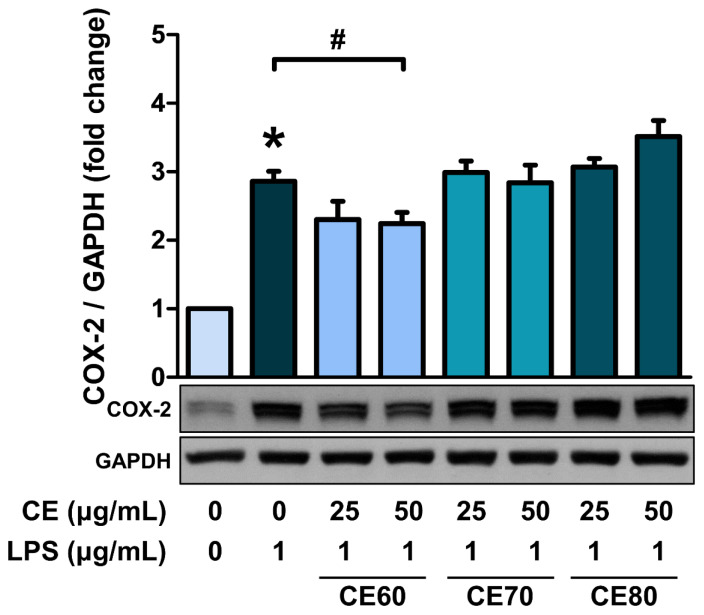
COX-2 expression in MAC-T cells treated with different solvent extracts of *Centella asiatica* (CE). Cells were pre-treated with CE60, CE70, and CE80 (25 and 50 μg/mL) for 12 h, followed by LPS treatment (1 μg/mL) for an additional 12 h. Western blot analysis was used to measure COX-2 expression levels in whole cell lysates. GAPDH was used as a loading control. The western blots shown are representative images of three independent experiments. All experiments were performed in triplicate (n = 3), and the data represent the mean±standard error. The extracts obtained using different solvents were named as follows: CE60 (extracted with 60% ethanol), CE70 (extracted with 70% ethanol), and CE80 (extracted with 80% ethanol). * indicates a significant difference compared to cells treated with 0 μg/mL of CE (p<0.05). # indicates a significant difference compared to cells treated with 1 μg/mL of LPS (p<0.05). COX-2, cyclooxygenase-2; GAPDH, glyceraldehyde 3-phosphate dehydrogenase; LPS, lipopolysaccharide.

COX-2 protein expression, an indicator of inflammation, was analyzed to identify the most effective CE in anti-inflammatory properties [27]. LPS significantly increased COX-2 protein expression in MAC-T cells, and pre-treatment of cells with CE70 and CE80 at 25 and 50 μg/mL did not inhibit the upregulation of COX-2 (Figure 4). However, pre-treatment of cells with CE60 at 50 μg/mL significantly suppressed the LPS-induced increase in COX-2 expression. Therefore, CE 60 was selected for further experiments.

**Figure 5:**
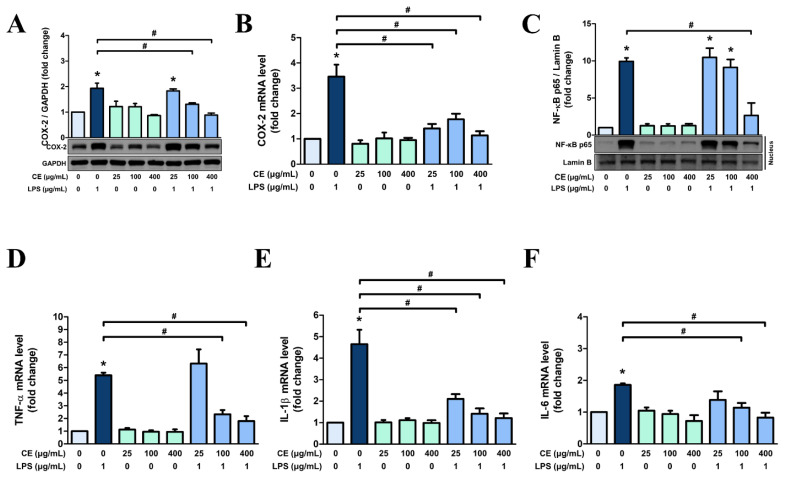
Anti-inflammatory effects of *Centella asiatica* extracted with 60% ethanol (CE60) on MAC-T cells. (A, B, D, E, and F) Cells were pre-treated with CE60 (25, 100, and 400 μg/mL) for 12 h, followed by LPS treatment (1 μg/mL) for 12 h. (A) Western blot analysis was used to measure protein levels of COX-2. GAPDH was used as a loading control. Gene expressions of (B) *COX-2*, (D) *TNF-α*, (E) *IL-1β*, and (F) *IL-6* were measured using RT-PCR. The mRNA expression levels were calculated relative to GAPDH expression. (C) Cells were pre-treated with CE60 at concentrations of 25, 100, and 400 μg/mL for 12 h, exposure to LPS at 1 μg/mL for 6 h. The nuclear translocation of NF-κB p65 was assessed using nuclear fractionation and Western blot analysis, with Lamin B serving as a loading control. All experiments were performed in triplicate (n = 3), and the data represent the mean±standard error. * indicates a significant difference compared to cells treated with 0 μg/mL of CE60 (p<0.05). # indicates a significant difference compared to cells treated with 1 μg/mL of LPS (p<0.05). COX-2, cyclooxygenase-2; GAPDH, glyceraldehyde 3-phosphate dehydrogenase; LPS, lipopolysaccharide; NF-κB, nuclear factor kappa B; TNF-α, tumor necrosis factor-α; IL-1β, interleukin-1 beta; IL-6, interleukin-6; RT-PCR, real-time polymerase chain reaction.

Further, protein and mRNA expressions of inflammation-related factors in MAC-T cells were evaluated using various concentrations of CE60 (0, 25, 100, and 400 μg/mL). Both the protein and mRNA expression levels of COX-2 were significantly increased in cells following LPS stimulation (Figures 5A, 5B). However, pre-treatment of cells with CE60 at 100 and 400 μg/mL attenuated the protein and mRNA expression of COX-2. Additionally, MAC-T cells treated with CE60 alone showed no significant difference in COX-2 expression compared to the control group.

Given that COX-2 expression is regulated by the NF-κB activation [28], nuclear translocation of the NF-κB subunit p65 was determined (Figure 5C). In parallel with COX-2 protein expression, the translocation of NF-κB p65 was enhanced with the LPS treatment. Cells treated with CE60 alone exhibited no significant differences compared to the control group. However, MAC-T cells pre-treated with 400 μg/mL of CE60 showed a significant reduction in LPS-induced nuclear translocation of NF-κB p65.

Furthermore, we examined the mRNA expressions of pro-inflammatory cytokines, *TNF-α*, interleukin-1 beta (*IL-1β*), and interleukin-6 (*IL-6*), crucial indicators of inflammatory responses [29]. The mRNA expressions of *TNF-α*, *IL-1β*, and *IL-6* that increased due to LPS were significantly decreased by pre-treatment of cells with CE60, particularly at concentrations of 100 and 400 μg/mL (Figures 5D–5F).

### High-performance liquid chromatography analysis of *Centella asiatica* extract with 60% ethanol

The bioactive properties of *C. asiatica* are primarily attributable to the presence of four triterpenes: madecassoside, asiaticoside, madecassic acid, and asiatic acid. To identify and quantify these bioactive compounds in CE60, we conducted HPLC analysis. The quantification of the four triterpenes in CE60 was based on the area of the chromatogram (Supplement 2). The standard retention times for madecassoside, asiaticoside, madecassic acid, and asiatic acid were 9.384 min, 11.413 min, 21.401 min, and 26.024 min, respectively. Among these compounds, asiaticoside was the most abundant in CE60, constituting 57.0348% of the total detected triterpenes, followed by madecassoside (25.4279%), asiatic acid (11.1916%), and madecassic acid (6.3457%). Therefore, our HPLC analysis suggests that asiaticoside is the major bioactive component of CE60.

### *Centella asiatica* extract with 60% ethanol reduces reactive oxygen species production in MAC-T cells

**Figure 6:**
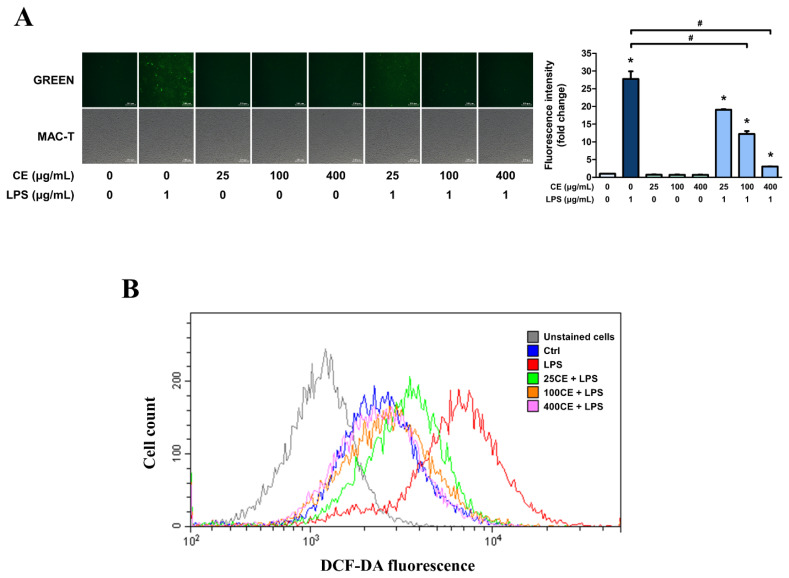
Effect of *Centella asiatica* extracted with 60% ethanol (CE60) on reactive oxygen species (ROS) production in MAC-T cells. Cells were pre-treated with CE60 (25, 100, and 400 μg/mL) for 12 h, followed by LPS treatment (1 μg/mL) for 4 h. The cells were stained with 2′,7′-dichlorofluorescin diacetate (DCFH-DA) to detect intracellular ROS production. (A) The intensity of green fluorescence (ROS production) was assessed using a fluorescence microscope. The DCFH-DA positive area (green fluorescence) was analyzed using Image J software. (B) Cells incubated with DCFH-DA were analyzed for ROS production using a CytoFLEX flow cytometer. In each treatment group, 25,000 cells were counted, and the DCFH-DA-positive cells were detected and analyzed using CytExpert software. All experiments were performed in triplicate (n = 3), and the data represent the mean±standard error. * indicates a significant difference compared to cells treated with 0 μg/mL of CE60 (p<0.05). # indicates a significant difference compared to cells treated with 1 μg/mL of LPS (p<0.05). LPS, lipopolysaccharide.

Intracellular ROS levels in MAC-T cells were measured using the fluorescent dye, DCFH-DA. Intracellular ROS oxidizes DCFH-DA, leading to the formation of a compound that emits green fluorescence. Consequently, the intensity of green fluorescence served as an indicator of intracellular ROS levels. Treatment of MAC-T cells with LPS significantly increased the ROS levels (Figure 6A). In addition, cells treated with CE60 alone exhibited no significant green fluorescence. However, MAC-T cells pre-treated with CE60 displayed a significant reduction in ROS production, even when stimulated with LPS. This suggests that CE60 at concentrations of 100 and 400 μg/mL suppressed intracellular ROS generation. The intracellular DCFH-DA levels were quantified using a CytoFLEX flow cytometer (Figure 6B). Increased ROS production was observed in MAC-T cells following LPS treatment. However, pre-treatment of cells with CE60 resulted in a concentration-dependent reduction in ROS production, indicating the ability of CE60 to mitigate ROS generation in MAC-T cells.

### *Centella asiatica* extract with 60% ethanol induces antioxidant enzymes in MAC-T cells

**Figure 7:**
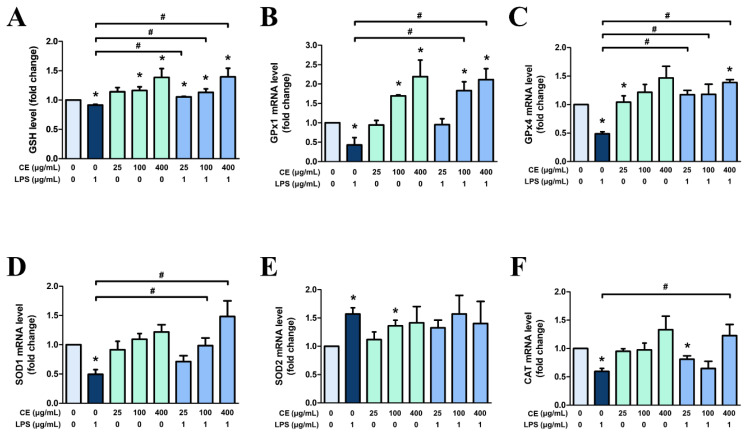
Anti-oxidative effects of *Centella asiatica* extracted with 60% ethanol (CE60) on MAC-T cells. Cells were pre-treated with CE60 (25, 100, and 400 μg/mL) for 12 h, followed by LPS treatment (1 μg/mL) for 12 h. (A) Glutathione (GSH) concentrations were quantified utilizing a fluorometric assay. The fluorescence intensity was assessed with a microplate reader. Gene expressions of (B) *GPx1*, (C) *GPx4*, (D) *SOD1*, (E) *SOD2*, and (F) *CAT* were measured using RT-PCR. mRNA expression levels were calculated relative to GAPDH expression. All experiments were performed in triplicate (n = 3), and the data represent the mean±standard error. * indicates a significant difference compared to cells treated with 0 μg/mL of CE60 (p<0.05). # indicates a significant difference compared to cells treated with 1 μg/mL of LPS (p<0.05). LPS, lipopolysaccharide; GPx, glutathione peroxidase; SOD, superoxide dismutase; CAT, catalase; RT-PCR, real-time polymerase chain reaction; GAPDH, glyceraldehyde 3-phosphate dehydrogenase.

Living organisms possess antioxidant systems that reduce intracellular ROS formation and promote ROS scavenging. Therefore, we examined the role of CE60 on the mRNA expression of enzymatic antioxidants (*GPx1*, *GPx4*, *SOD1*, *SOD2*, and *CAT*) and the non-enzymatic antioxidant GSH, all of which are involved in the antioxidant process (Figure 7). When MAC-T cells were treated solely with LPS, the levels of GSH and mRNA expression of *CAT*, *GPx1*, *GPx4*, and *SOD1* were significantly reduced, whereas *SOD2* increased (Figure 7). Pre-treatment of cells with CE60 led to a concentration-dependent increase in GSH levels and the mRNA expression of *GPx1*, *GPx4*, *SOD1*, and *CAT* (Figure 7). Pre-treatment of cells with 400 μg/mL of CE60 followed by LPS stimulation resulted in increased GSH levels and elevated mRNA expression of antioxidant enzymes, except for *SOD2*. In the case of *SOD2*, 400 μg/mL of CE60 recovered the mRNA levels of the overexpressed *SOD2* to a level like that of the control. This suggests that CE60 enhances the antioxidant ability of MAC-T cells, thereby attenuating the formation or accumulation of intracellular ROS.

### *Centella asiatica* extract with 60% ethanol induces antioxidant enzymes in MAC-T cells through activating nuclear factor erythroid-2-related factor 2

**Figure 8:**
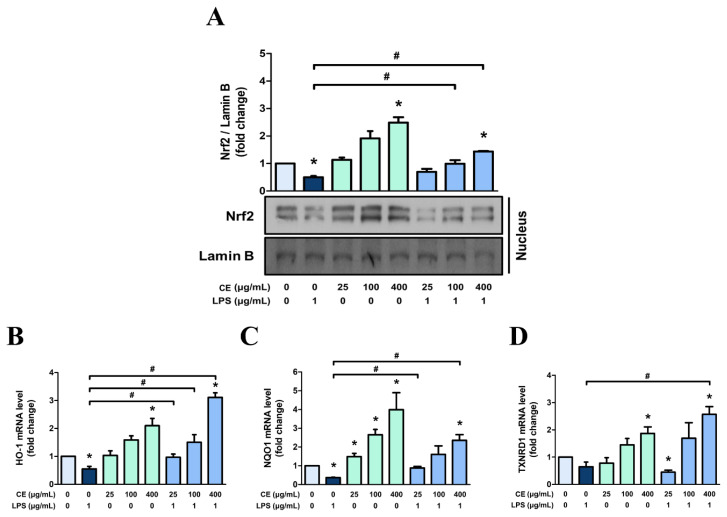
Effect of *Centella asiatica* extracted with 60% ethanol (CE60) on nuclear translocation of Nrf2 and related gene expressions in MAC-T cells. (A) Cells were pre-treated with CE60 at concentrations of 25, 100, and 400 μg/mL for 12 h, followed by LPS treatment at 1 μg/mL for 6 h. The nuclear translocation of Nrf2 was evaluated using nuclear fractionation and Western blot analysis, with Lamin B used as a loading control. (B–D) Cells were pre-treated with CE60 at at concentrations of 25, 100, and 400 μg/mL for 12 h, followed by LPS treatment at 1 μg/mL for 12 h. Gene expressions of (B) *HO-1*, (C) *NQO1*, and (D) *TXNRD1* were measured using RT-PCR. mRNA expression levels were calculated relative to GAPDH expression. All experiments were performed in triplicate (n = 3), and the data represent the mean±standard error. * indicates a significant difference compared to cells treated with 0 μg/mL of CE60 (p<0.05). # indicates a significant difference compared to cells treated with 1 μg/mL of LPS (p<0.05). Nrf2, nuclear factor erythroid-2-related factor 2; LPS, lipopolysaccharide; HO-1, heme oxygenase-1; NQO1, NAD(P)H quinone oxidoreductase 1; TXNRD1, thioredoxin reductase 1; RT-PCR, real-time polymerase chain reaction; GAPDH, glyceraldehyde 3-phosphate dehydrogenase.

Nrf2 serves as the central mediator of the antioxidant system, and its activation involves translocation to the nucleus, thereby facilitating the transcription of genes encoding antioxidant response elements (ARE), including NAD(P)H quinone oxidoreductase 1 (NQO1), heme oxygenase-1 (HO-1), and thioredoxin reductase 1 (TXNRD1). In cells treated with LPS only, the nuclear translocation of Nrf2 was significantly decreased compared to that in the control group (Figure 8A). However, pre-treatment of cells with 400 μg/mL of CE60 substantially induced the translocation of Nrf2, compared to LPS-treated cells (Figure 8A).

Moreover, LPS decreased the mRNA expression of *HO-1* and *NQO1* in MAC-T cells (Figures 8B, 8C). However, pre-treatment of cells with CE60 led to a concentration-dependent increase in the mRNA levels of *HO-1*, *NQO1*, and *TXNRD1* (Figures 8B–8D). When MAC-T cells were pre-treated with 400 μg/mL of CE60 followed by LPS stimulation, the mRNA levels of these antioxidant enzymes significantly increased compared to MAC-T cells treated only with LPS (Figures 8B–8D). These findings suggest that 400 μg/mL of CE60 can induce the phase II detoxification enzymes in MAC-T cells through the activation of Nrf2 translocation.

### Effects of *Centella asiatica* extract with 60% ethanol on casein synthesis in differentiated bovine mammary epithelial cells

**Figure 9:**
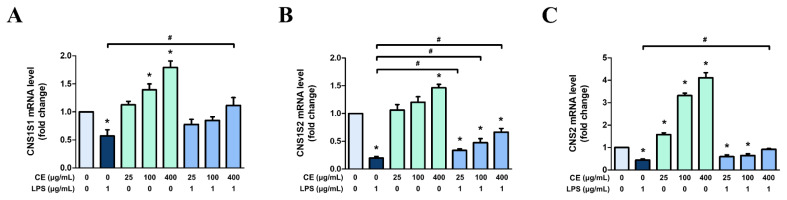
Effect of *Centella asiatica* extracted with 60% ethanol (CE60) on casein synthesis in differentiated MAC-T cells. Cells were differentiated for 8 days and pre-treated with CE60 (25, 100, and 400 μg/mL) for 12 h, followed by LPS treatment (1 μg/mL) for 12 h. Gene expressions of (A) *CSN1S1*, (B) *CSN1S2*, and (C) *CSN2* were measured using RT-PCR. mRNA expression levels were calculated relative to GAPDH expression. All experiments were performed in triplicate (n = 3), and the data represent the mean±standard error. * indicates a significant difference compared to cells treated with 0 μg/mL of CE60 (p<0.05). # indicates a significant difference compared to cells treated with 1 μg/mL of LPS (p<0.05). LPS, lipopolysaccharide; RT-PCR, real-time polymerase chain reaction; GAPDH, glyceraldehyde 3-phosphate dehydrogenase.

To assess the potential effect of CE60 on milk casein protein synthesis, the gene expression levels of casein isoforms were analyzed in differentiated MAC-T cells. Quantitative RT-PCR was used to evaluate the mRNA expression levels of α-casein S1 (*CSN1S1*), α-casein S2 (*CSN1S2*), and β-casein (*CSN2*). Following LPS treatment, the mRNA expression levels of the three casein isoforms (*CSN1S1*, *CSN1S2*, and *CSN2*) were significantly decreased (Figure 9). In contrast, CE60 restored *CSN1S1* gene expression levels to those of the control (Figure 9). Importantly, casein gene expression in differentiated cells was significantly increased by CE60 treatment alone at concentrations of 25, 100, and 400 μg/mL compared to the control (Figures 9A–9C). These results suggest that CE60 alone can increase casein synthesis under both normal and LPS-induced inflammatory conditions.

## DISCUSSION

Bovine mastitis is a common disease in dairy cows around parturition, primarily caused by bacterial infection such as *Escherichia coli* [27,30]. In mammary epithelial cells, bacterial infection triggers immune responses through the recognition of LPS, a component of the bacterial outer membrane, thereby promoting the production of ROS and inducing acute inflammatory responses [27,31]. However, excessive ROS generation disrupts the balance between cellular oxidants and reductants, leading to OS [31]. Natural antioxidant additives have gained attention for their beneficial effects without side effects. *C. asiatica* has been reported to possess antioxidant and anti-inflammatory properties [32]. Consequently, we used CE on LPS-treated bovine mammary epithelial cells to investigate its protective effects against OS and inflammation in the context of bovine mastitis.

Different ethanol concentrations (60%–100%) and DW were used to extract *C. asiatica*. TPC, free radical scavenging activity, and anti-inflammatory effects were assessed. CE extracted with 60%, 70%, and 80% ethanol (CE60, CE70, CE80) showed higher DPPH and ABTS+ scavenging activity and TPC. Lower ethanol concentrations increased solvent polarity, extracting more phenolic compounds [33]. These extracts also demonstrated strong radical scavenging due to high phenolic content. Cell viability and LDH release assays showed CE60, CE70, and CE80 were not cytotoxic. CE60 exhibited the strongest anti-inflammatory effect by attenuating LPS-induced COX-2 expression [22]. Therefore, CE60 was selected for further study.

HPLC analysis of CE60 revealed asiaticoside as the most abundant triterpene, followed by madecassoside, asiatic acid, and madecassic acid [10]. These triterpenes are known for bioactivity of *C. asiatica*. The study examined the effects of CE60 on pro-inflammatory markers (COX-2, TNF-α, IL-1β, IL-6) and NF-κB, a key transcription factor in inflammation [34]. LPS increased NF-κB p65 mRNA expression, which CE60 pre-treatment reduced. Similarly, CE60 attenuated LPS-induced increases in COX-2, TNF-α, IL-1β, and IL-6 mRNA levels. CE60 alone did not affect these markers. These results suggest triterpenes of CE60, particularly asiaticoside, contribute to its anti-inflammatory effects by downregulating NF-κB [35].

While ROS kills bacteria, an imbalance between ROS and antioxidants causes OS and tissue damage, contributing to inflammatory diseases like mastitis in dairy cattle [36]. Enhancing antioxidant activity is crucial. CE60 pre-treatment decreased LPS-induced intracellular ROS accumulation in a concentration-dependent manner, without inducing ROS production itself. Cells employ enzymatic (SOD, CAT, GPx) and non-enzymatic (GSH, ascorbic acid) antioxidant systems [37]. CE60 increased intracellular GSH levels, protecting against LPS-induced GSH depletion [38]. CE60 also upregulated GPx1 and GPx4 mRNA expression, restoring LPS-suppressed levels. While SOD2 mRNA increased with LPS, likely due to a cellular counter-response [39], CE60 did not significantly affect CAT or SOD1 mRNA [40]. These findings suggest CE60 boosts both enzymatic and non-enzymatic antioxidant systems. Cells also activate defense pathways like Nrf2 [41]. CE60 activated Nrf2 by increasing its nuclear translocation and reversing LPS-induced Nrf2 reduction. CE60 also increased HO-1, NQO1, and TXNRD1 mRNA expression, similar to Nrf2, protecting against LPS [42]. These enzymes are involved in phase II detoxification. These effects are likely due to triterpenes and phenolic acids in CE [43].

Finally, the effect of CE on milk protein synthesis was demonstrated by evaluating the expression of casein genes (*CSN1S1*, *CSN1S2*, and *CSN2*) in differentiated MAC-T cells [23,24,44]. Pre-treatment of cells with CE protected the LPS-induced suppression of casein gene expression, indicating its preventive role against OS-related impairment of milk protein synthesis. *C. asiatica* is generally recognized as safe and has been widely used in cosmetics and dermatological treatments for skin health and wound healing [45,46]. Recent studies have reported that supplementation with *C. asiatica* extract or powder as a feed additive can enhance rumen fermentation and digestibility as a feed additive in ruminants, owing to its antiprotozoal, antibacterial, and antioxidant properties [47,48]. Considering previous reports, our data suggest CE has potential to counteract the LPS-induced decline in milk production due to mastitis in dairy cows, whether given orally or topically.

## CONCLUSION

In summary, the extraction of *C. asiatica* using 60% ethanol exhibited the highest TPC and free radical scavenging activity. LPS treatment in MAC-T cells led to OS, inflammation, and decreased milk productivity. However, CE60 pre-treatment effectively counteracted LPS-induced OS and inflammatory responses while restoring milk productivity. While more animal studies are needed to confirm the protective effects of CE60 and its potential as a feed additive or topical treatment for bovine mastitis, our findings suggest that *C. asiatica* is a promising natural agent for preventing the condition in dairy cows.

## SUPPLEMENTARY MATERIAL

**Supplement 1**. Primer sequences used in quantitative real-time PCR.

**Supplement 2**. Triterpene concentration of CE60.
