# Supplementary material for: Protection of bovine mammary epithelial cells against lipopolysaccharide-induced inflammatory responses using Centella asiatica through its antioxidant and anti-inflammatory activities
Source: Anim Biosci. 2025 Aug 12;38(11):2403–17. doi: 10.5713/ab.25.0089 (PMC12580958; doi:10.5713/ab.25.0089)
Supplement: Supplementary file 1 [file ab-25-0089-supplementary-1.pdf]

SUPPLEMENTARY MATERIALS

Supplement 1. Primer sequences used in quantitative real-time PCR

Name of genes	Accession number	Primer sequence (5'-3')
TNF-α[1)]	EU276079	(F) ACG GGC TTT ACC TCA TCT ACT CAC<br>(R) TTG ACC TTG GTC TGG TAG GAG ACT
IL-6	EU276071	(F) AGC GCA TGG TCG ACA AAA TCT C<br>(R) AAC CCA GAT TGG AAG CAT CCG T
IL-1β	EU276067	(F) CCG TAC CTG AAC CCA TCA ACG AAA<br>(R) GGT GTT GGA TGC AGC TCT TCA TCT
COX-2	AF004944	(F) GCA TTC TTT GCC CAG CAC TTC A<br>(R) TCC ACC CCA TGG TTC TTT CCC TTA
HO-1	BT020968	(F) AGG ATT TGT CAG AGG CCC TGA A<br>(R) CAA AGA CGC CAT CAC CAG CTT A
NQO1	NM_001034535	(F) GGT GCT CAT AGG GGA GTT CG<br>(R) GGG AGT GTG CCC AAT GCT AT
TXNRD1	NM_174625	(F) CGG TAT TGC TGG CAA TAG GAA GAG<br>(R) GGC ATA GAT GTA AGG CAC GTT GGT
Gpx1	NM_174076	(F) GCT CAT TGA GAA CGT AGC ATC G<br>(R) TTT CCT GAT GCC CAA ACT GGT
Gpx4	NM_001346430	(F) CTG TGC TCG CTC CAT GCA CGA AT<br>(R) CCT CAT TGC GAG GCC ACA TTG GTG
SOD1	NM_174615	(F) CGG TGC AAG GCA CCA TCC ACT TC<br>(R) GAG GAC CTG CAC TGG TAC AGC CT
SOD2	BT020988	(F) AAT CTG AGC CCT AAC GGT GG<br>(R) GTA AGC GTC CCT GCT CCT TA
CAT	NM_001035386	(F) GCT CCA AAT TAC TAC CCC AAT AGC<br>(R) GCA CTG TTG AAG CGC TGT ACA
CNS1S1	NM_181029	(F) ACT GAG GAT CAA GCC ATG GAA G<br>(R) GAA TGT GCT TCT GCT CAA CAC T
CNS1S2	NM_174528	(F) CTG GAA TTA ACT GCT TCT ACC T<br>(R) TAC TCT GCG ATT TGT CTT ATT GA
CNS2	M55158	(F) AGC CTC TTC CTC CAA CTG TC<br>(R) ATC TCT CTG GGG ATA GGG CA
GAPDH	NM_001034034	(F) ATG ATT CCA CCC ACG GCA AGT T<br>(R) ACC ACA TAC TCA GCA CCA GCA T

[1)]TNF-α, tumor necrosis factor alpha; IL-6, interleukin 6; IL-1β, interleukin 1 beta; COX-2, cyclooxygenase-2; HO-1, heme oxygenase 1; NQO1, nicotinamide adenine dinucleotide phosphate oxygenase 1; TXNRD1, thioredoxin reductase 1; Gpx1, glutathione peroxidase 1; Gpx4, glutathione peroxidase 4; SOD1, superoxide dismutase 1; SOD2, superoxide dismutase 2; CAT, catalase; CNS1S1, α-casein S1; CNS1S2, α-casein S2; CNS2, β-casein; GAPDH, glyceraldehyde 3-phosphate dehydrogenase.
