# Supplementary material for: Protection of bovine mammary epithelial cells against lipopolysaccharide-induced inflammatory responses using Centella asiatica through its antioxidant and anti-inflammatory activities
Source: Anim Biosci. 2025 Aug 12;38(11):2403–17. doi: 10.5713/ab.25.0089 (PMC12580958; doi:10.5713/ab.25.0089)
Supplement: Supplementary file 2 [file ab-25-0089-supplementary-2.pdf]

Supplement 2. Triterpene concentration of CE60

Types of triterpenes	Retention time (min)	Concentration of triterpenes in CE60[1)] (mg/mL)
Madecassoside	9.384	25.073
Asiaticoside	11.413	59.678
Madecassic acid	21.401	2.609
Asiatic acid	26.024	4.325

[1)] *Centella asiatica* extract using 60% ethanol solution.
